# TREX1 is expressed by microglia in normal human brain and increases in regions affected by ischemia

**DOI:** 10.1111/bpa.12626

**Published:** 2018-10-10

**Authors:** Parul H. Kothari, Grant R. Kolar, Joanna C. Jen, Rula Hajj-Ali, Paula Bertram, Robert E. Schmidt, John P. Atkinson

**Affiliations:** 1 Department of Biology and Biomedical Sciences Human & Statistical Genetics Program, Washington University School of Medicine, St. Louis, MO.; 2 Division of Rheumatology, Immunology and Allergy, Department of Medicine, Brigham and Women’s Hospital, Harvard Medical School, Boston, MA.; 3 Channing Division of Network Medicine, Department of Medicine, Brigham and Women’s Hospital, Harvard Medical School, Boston, MA.; 4 Department of Pathology & Immunology, Washington University School of Medicine, St. Louis, MO.; 5 Department of Pathology and Department of Ophthalmology, Saint Louis University School of Medicine, St. Louis, MO.; 6 Departments of Neurology and Neurobiology, UCLA School of Medicine, Los Angeles, CA.; 7 Departments of Neurology, Otolaryngology, Neurosurgery, Icahn School of Medicine at Mount Sinai, New York, NY.; 8 Center for Vasculitis Care and Research, Cleveland Clinic Lerner College of Medicine, Orthopaedic and Rheumatologic Institute, Cleveland, OH.; 9 Department of Medicine, Division of Rheumatology, Washington University School of Medicine, St. Louis, MO.; 10 Department of Pathology and Immunology, Division of Neuropathology, Washington University School of Medicine, St. Louis, MO.

**Keywords:** brain ischemia, DNase1, macrophages, microglia, TREX1, vasculopathy

## Abstract

**Background:**

Mutations in the three-prime repair exonuclease 1 (TREX1) gene have been associated with neurological diseases, including Retinal Vasculopathy with Cerebral Leukoencephalopathy (RVCL). However, the endogenous expression of TREX1 in human brain has not been studied.

**Methods:**

We produced a rabbit polyclonal antibody (pAb) to TREX1 to characterize TREX1 by Western blotting (WB) of cell lysates from normal controls and subjects carrying an RVCL frame-shift mutation. Dual staining was performed to determine cell types expressing TREX1 in human brain tissue. TREX1 distribution in human brain was further evaluated by immunohistochemical analyses of formalin-fixed, paraffinembedded samples from normal controls and patients with RVCL and ischemic stroke.

**Results:**

After validating the specificity of our anti-TREX1 rabbit pAb, WB analysis was utilized to detect the endogenous wild-type and frame-shift mutant of TREX1 in cell lysates. Dual staining in human brain tissues from patients with RVCL and normal controls localized TREX1 to a subset of microglia and macrophages. Quantification of immunohistochemical staining of the cerebral cortex revealed that TREX1^+^ microglia were primarily in the gray matter of normal controls (22.7 ± 5.1% and 5.5 ± 1.9% of Iba1^+^ microglia in gray and white matter, respectively) and commonly in association with the microvasculature. In contrast, in subjects with RVCL, the TREX1^+^ microglia were predominantly located in the white matter of normal appearing cerebral cortex (11.8 ± 3.1% and 38.9 ± 5.8% of Iba1^+^ microglia in gray and white matter, respectively). The number of TREX1^+^ microglia was increased in ischemic brain lesions in central nervous system of RVCL and stroke patients.

**Conclusions:**

TREX1 is expressed by a subset of microglia in normal human brain, often in close proximity to the microvasculature, and increases in the setting of ischemic lesions. These findings suggest a role for TREX1^+^ microglia in vessel homeostasis and response to ischemic injury.

## INTRODUCTION

The autosomal dominant disease Retinal Vasculopathy with Cerebral Leukoencephalopathy (RVCL) is characterized by a systemic vasculopathy and encompasses the disorders reported separately as Cerebroretinal Vasculopathy (CRV), Hereditary Vascular Retinopathy (HVR), and Hereditary Endotheliopathy with Retinopathy, Nephropathy, and Stroke (HERNS). (6,11,24,26,30) RVCL is 100% penetrant by middleage when symptoms first develop. Visual defects due to peri-foveal capillary drop-out are typically followed by focal and global neurological deficits as well as cognitive decline due to white matter lesions in the cerebral hemispheres and cerebellum. Neuroimaging from patients with RVCL shows punctate, hyperintense lesions in almost all affected patients with the majority also developing rim-enhancing mass lesions, often associated with edema, as the disease progressed. (26) Although the retina and white matter are most prominently affected, other organs including the liver and kidney show histopathological changes attributable to small vessel disease. All patients endure an early death from neurological decline due to progressive white matter lesions. The vasculopathy is characterized by vascular wall thickening, multilaminated basement membranes, and luminal narrowing that can progress to complete obliteration. The decrease in blood flow results in ischemic necrosis and symptomatology, particularly in the retina and white matter.

This devastating disease is caused by frame-shift mutations in *TREX1*, the three prime repair exonuclease 1, that result in loss of its carboxy-tail. A truncated protein is synthesized and secreted but is lacking the site for its attachment to the endoplasmic reticulum (8,33). Consequently, the protein becomes mislocated, being observed in the cytoplasm and nucleus (24). While 14 different frame-shift mutations have been identified, the most common is the V235fs seen in 13 unrelated kindreds. (26,31) All of the RVCL-associated mutations lead to a functional exonuclease distributed diffusely in the cytoplasm and nucleus instead of being anchored to the endoplasmic reticulum (ER) like the wild-type protein.(24)

In a prior report (Supplementary Table 3) (26), we described 11 families in carrying five different mutations (6/11 V235fs and 2/11 T249fs). By history and genetic analyses, these families were not related (26). The results are most consistent with the mutations being independent events with a relative “hot spot” at V235fs site. Since publication of this article in 2016, we have now identified (as of 04/01/18—M.K. Liszewski and J.P. Atkinson, unpublished data) a total of 26 families worldwide in which 13 carry the V235fs variant and 13 others have fs variants corresponding to amino acid positions from 236 to 310 in the mature TREX1 protein. Most of the families are of European origin including two of Ashkenazi-Jewish ethnicity and one family of Chinese background in Taiwan.

With respect to the types of mutations in RVCL compared to those in AGS, SLE, and FCL, the RVCL mutations are frame-shift mutations in the tail, while those in AGS, SLE, and FCL are mostly missense or nonsense and primarily in the enzymatic amino-terminal portion of the protein. For readers interested in the various mutations in these other diseases, we refer the reader to two recent reviews.(14,26,33)

The physiologic role of TREX1 is incompletely understood but recent studies have supported a role in clearing single stranded DNA (ssDNA) derived from endogenous retroelements.(29,35) In the absence of functional TREX1, ssDNA accumulates and triggers an antiviral state with the induction of interferon (IFN) stimulated genes.(5) The knock out mouse develops an inflammatory myocarditis resulting in death at 2–6 months.(19,29) As such, TREX1 is thought to play a physiologic role in preventing autoimmunity.

These findings are relevant for three other human diseases associated with *TREX1* mutations, Aicardi- Gouiteres syndrome (AGS), systemic lupus erythematosus (SLE), and familial chilblain lupus (FCL).(3,13,14,20) In each of these diseases, *TREX1* mutations usually decrease or abolish exonuclease function. AGS mimics a congenital viral infection of the brain and typically manifests as a neurodevelopmental disorder in early infancy. As the extra-neurological findings in AGS overlap with SLE, AGS has been proposed as a genetic model of systemic autoimmunity.(14) RVCL though does not clinically or pathologically resemble an autoimmune disease but rather a premature degeneration of the microvasculature.(26)

Most studies have focused on the exonuclease activity of TREX1, for which the carboxy-terminus is not necessary. Recently, it has been shown that the carboxy-terminus plays a role in suppressing glycan-induced inflammation, a function that is lost by the frame-shift mutations of RVCL.(8) More recently, mice carrying a common RVCL frame-shift mutation have developed autoantibodies although this is infrequently observed in patients with RVCL.(15,26) However, the mechanism by which this mislocalized but enzymatically active exonuclease results in a systemic vasculopathy remains elusive.

Although TREX1 transcripts have been identified at the RNA level in all tissues examined, the endogenous expression of the protein has not been studied.(17) Given the burden of the neurological disease in RVCL, we evaluated the expression of TREX1 in human brain tissue using a rabbit polyclonal antibody (pAb) we produced. With the use of immunohistochemical and immunofluorescent staining, we sought to identify the specific cell types expressing TREX1 in the brain for insight into the relationship between this exonuclease and the microvasculature.

## MATERIALS AND METHODS

### Antibody production

As insufficient amounts of recombinant wild-type TREX1 could be recovered by us (unpublished) or others,(10,16,17) recombinant V235fs mutant TREX1 was produced in *E. coli* (detailed in the Supplementary Methods).

### Antibody purification

The rabbit pAb to TREX1 was purified by precipitation with saturated ammonium sulfate for use in WB analyses. For immunofluorescence and immunohistochemical staining, the pAb was further purified by antigen affinity to the recombinant *E. coli* immunogen (V235fs). Details are supplied in the Supplementary Methods. The purified Ab preparations were electrophoresed on a Tris-glycine gel and stained with Coomassie Blue to assess purity. Preincubation of 1 μg/ml Ab with 10 μg/ml recombinant *E. coli* heat shock protein 70 (Hsp70, also known as DnaK; ProSpec, Ness Ziona, Israel) was accomplished at 4°C for 3 h with rotation.

### Plasmid construct, cell culture, and transfection

**Figure 1.**
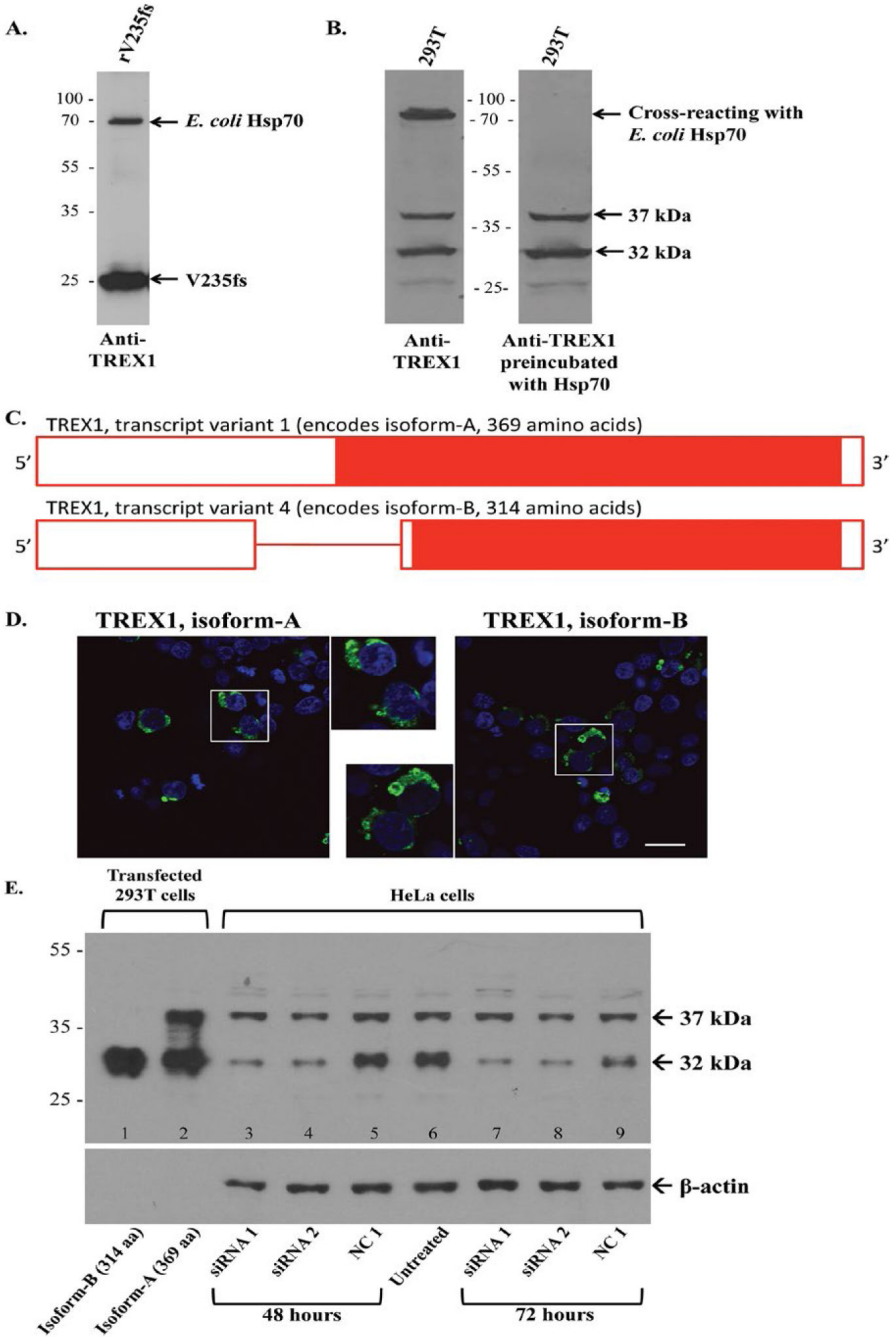
Expression and detection of TREX1 proteins. **A.** The rabbit anti-TREX1 Ab detects the recombinant TREX1 (rV235fs) as well as *E. coli* heat shock protein 70 (Hsp70) in the immunogen on Western blot (WB). **B.** On strips from the same WB, the anti-TREX1 Ab detects a band at ∼75 kDa in 293T cells (left panel) that is eliminated after preincubation with *E. coli* Hsp70. The bands at 37 and 32 kDa are unchanged. **C.** A schematic of TREX1 transcript variants with solid red bar for coding sequence, open red bar for untranslated regions, and red line for intron. Transcript variant 1 retains the intron and encodes a longer N-terminus. **D.** 293T cells transiently transfected with fluorescent protein (FP)-tagged wild-type TREX1 (green) for isoforms-A (left panel) and B (right panel). Nuclei are counterstained with TO-PRO-3 (blue). Scale bar represents 28 μm. **E.** Representative WB of TREX1 from 293T cells transfected with wild-type TREX1 isoforms-B (lane 1) and A (lane 2) for positive controls and HeLa cells transfected with TREX1 siRNA 1 (lanes 3 and 7) or 2 (lanes 4 and 8), negative control (NC1, lanes 5 and 9) as well as an untreated control (lane 6). Cell lysates were evaluated at 48 (lanes 3–5) and 72 (lanes 7–9) h after transfection with siRNA. Membrane was stripped and reprobed with anti-β-actin Ab as a loading control.

The cDNA for human TREX1 was obtained from OriGene Technologies (Rockville, MD) and variants 1 and 4 (Fig. 1) were cloned into the EcoR1/XbaI sites in the pSBC expression vector, with and without an amino-terminal fluorescent protein (FP) tag. The frame-shift mutation was introduced by site-directed mutagenesis (QuikChange II Site-Directed Mutagenesis Kit; Agilent, Santa Clara, CA).

The TREX1-specific siRNAs were targeted to the following sequences, 5’-GGTTGTGACAGCAGATGGTCTTGGCT-3’ (siRNA1) and 5’-CAAGGAAGAGCTACAGC CTAGGC AG-3’ (siRNA2). A universal negative control (NC1) was obtained from Integrated DNA Technologies (Coralville, IA).

Details on transfection of 293T cells with plasmid expression vectors and HeLa cells with siRNA can be found in the Supplementary Methods.

EBV transformed B lymphocytes were generated from normal controls and subjects carrying the RVCL mutation.(8,24)

### PCR and reverse transcription (RT)-PCR

Genomic DNA (gDNA) and RNA were obtained from cell lines using the Wizard Genomic DNA purification kit (Promega, Madison, WI) and RNeasy Mini Kit (Qiagen, Valencia, CA), respectively. RNA was treated with DNase I (Invitrogen) to remove DNA contamination. RNA was amplified with the Invitrogen SuperScript One-Step RT-PCR with Platinum Taq. The gDNA and RNA were amplified with Platinum Taq (Invitrogen). The forward and reverse primers to detect TREX1 transcript variants 1 and 4 were 5’-AGCGAGAGCCGCGGGAGAGTG-3’ and 5’-CACACGCGGTGGTGGAGGAA-3’. The forward and reverse primers to amplify TREX1 transcript variant 1 were 5’-GCCTGCTACGGTGAGTGTGGC-3’ and 5’-CTGCTGAGCCTGGAGAGGAGC-3’. PCR and RT-PCR were performed at 50°C for 30 min to allow reverse transcription and followed by 35 cycles at 94°C for 15 s, 60°C for 30 s and 72°C for 1 min. The resulting products were run on an agarose gel.

### Western blotting

Briefly, cell lysates were electrophoresed on 12% Tris-glycine gels (Life Technologies, Carlsbad, CA), transferred to a nitrocellulose membrane and blocked overnight in 5% nonfat dry milk in Tris-Buffered Saline and Tween 20 (TBS-T). The membrane was incubated with the primary Ab, rabbit pAb to TREX1 diluted 1:1000 or mouse monoclonal anti-β-actin Ab (Sigma Aldrich, St. Louis, MO) diluted 1:5000 in 5% non-fat dry milk. After washes with TBS-T, the membrane was incubated with the appropriate horseradish peroxidase (HRP)-conjugated secondary Ab followed by additional washes with TBS-T. The Western blots (WB) were developed with West Pico SuperSignal (Thermo Fisher Scientific, Waltham, MA). The protein bands from WB were scanned and quantified using ImageJ (NIH, Bethesda, MD).

### Immunofluorescence

293T cells were plated in 6-well chamber slides 24 h prior to transfection. Cells were transiently transfected (TransIT-293 transfection reagent; Mirus, Madison, WI) for 24 h before being fixed and permeabilized with acetone. Nuclei were counterstained with TO-PRO-3 (Life Technologies, Grand Island, NY). Cells were then visualized with a confocal microscope (Zeiss LSM510 META Laser Scanning Confocal Microscope; Carl Zeiss Microscopy, Jena, Germany).

Frozen sections were fixed with 95% ethanol, 5% glacial acetic acid for 30 min and then washed with PBS. Formalin-fixed, paraffin-embedded (FFPE) sections were hydrated sequentially with 5-min washes in xylene × 2, 95% ethanol × 2, 70% ethanol, 50% ethanol, and PBS. Antigen retrieval was performed in a pressure cooker with 1 mM EDTA pH 8 buffer for 2 min. After 3 PBS washes, the slides were blocked with avidin and biotin solutions (Vector Laboratories, Burlingame, CA). Slides were incubated with blocking buffer (10% serum diluted in PBS with 1% BSA) for 30 min and then with the primary Ab diluted in blocking buffer overnight at 4°C in a humidified chamber. Human serum was included in all blocking buffers to reduce non-specific background. After PBS washes, the slides were incubated for 1 h with an appropriate fluorophore-conjugated secondary Ab diluted in blocking buffer. The slides were washed with PBS and then incubated with TO-PRO-3 diluted 1:200 in PBS for 30 min. After PBS washes, coverslips were placed on slides using VectaMount Hard Set Fluorescent Mounting Media (Vector Laboratories) before visualization by confocal microscopy.

The primary Abs used included the rabbit pAb to TREX1 (1:200, 1 μg/ml), mouse monoclonal anti-CD68 (KP1 at 1:200; Abcam, Cambridge, MA), mouse monoclonal anti-MHC II (CR3/43 to HLA-DR-DP-DQ at 1:200; Abcam), mouse monoclonal anti-alpha smooth muscle actin (1A4 at 1:50; Abcam), mouse monoclonal anti-glial fibrillary acidic protein (GFAP; 1:200; Sigma) and biotinylated ricinus communis agglutinin I (RCA-1; Vector Laboratories). For slides stained with biotinylated RCA-1, blocking buffer was excluded and the primary Ab was diluted 1:10,000 in PBS and the FITC-Avidin secondary was diluted 1:200 in PBS. Secondary Abs alone were used as negative controls.

### Immunohistochemistry

FFPE sections underwent hydration, antigen retrieval, avidin-biotin blocking, and incubation with blocking buffer and primary and secondary Abs as described above. Endogenous peroxidase was quenched with a 3% solution of hydrogen peroxidase for 30 min prior to antigen retrieval. For slides stained with rabbit pAb to the ionized calciumbinding adapter molecule 1 (Iba1; Wako, Richmond, VA) diluted 1:1000 in blocking buffer, the secondary was a biotinylated anti-rabbit IgG (Jackson ImmunoResearch Laboratories, West Grove, PA). The signal on these slides was amplified with the Avidin-Biotin-HRP complex (ABC; Vector Laboratories) before being developed with the 3,3’-diaminobenzidine tetrachloride (DAB) substrate (Vector Laboratories) as the chromogen. For slides stained with rabbit pAb to TREX1 (diluted 1:200 in blocking buffer), the secondary was a peroxidase-conjugated anti-rabbit IgG (Jackson ImmunoResearch Laboratories). These slides were developed with DAB. Coverslips were mounted with VectaMount Permanent Mounting Media (Vector Laboratories).

### Experimental design

Serial sections stained with TREX1 or Iba1 were blinded and three sets of 10 consecutive images were obtained with a 40X objective for each brain region. Images were taken from the cerebral white and gray matter, basal ganglia, thalamus, hippocampus, and cerebellar molecular layer and white matter. All cell counts were confined to undamaged tissue. The total number of cells expressing TREX1 and Iba1 were counted for each image. A cell was considered to be within a given image only if the nucleus was present to prevent counts in two consecutive images. The average of all three sets was used for the data analysis. For each individual, the percent of TREX1^+^ microglia was calculated relative to the total number of Iba1^+^ cells in that section. The percent of Iba1^+^ cells in the white matter of the cerebral cortex was calculated relative to the total number of Iba1^+^ cells within the white and gray matter of that section for each subject. Similarly, the percent of TREX1^+^ cells in the white matter is reported relative to the total number of TREX1^+^ cells on that section.

### Study subjects

Tissue sections from the frontal lobe, occipital lobe, basal ganglia, hippocampus, thalamus, and cerebellum were obtained from all subjects except one case of RVCL (Table 1). In cases with RVCL or other neurological diseases, additional sections were available. The six normal controls were screened for the absence of neuropathology on hematoxylin and eosin (H&E) staining.

The five RVCL cases were selected based on the availability of tissue sections for analysis. All had their diagnoses well established at the time of death, including a known family history, a frame-shift mutation in *TREX1* and clinical manifestations of the characteristic white matter lesions and retinal vasculopathy.(26) Except for one case of RVCL from the Cleveland Clinic with the T249fs mutation, other subjects carried the V235fs mutation. They were from two unrelated individuals who underwent complete autopsies by the Department of Pathology and Immunology at Washington University School of Medicine. Tissue sections from all brain regions were available for four of the five RVCL cases. In one of the cases only the frontal lobe and additional sections from the cerebral cortex were available.(12)

At least two cases with radiation necrosis, multiple sclerosis, hemorrhagic stroke, and ischemic stroke were selected based on a search of the pathology database at the Washington University School of Medicine. A neuropathologist (GRK) selected the normal controls and cases. All tissue sections were de-identified prior to staining. The clinical demographics and pathological details of all subjects are presented in Table 1.

### Statistical analysis

In WB analyses, levels of TREX1 were adjusted relative to β-actin levels and are reported as mean ± standard deviation (SD). For quantification of immunohistochemical staining (detailed in Experimental design above), all data are presented as mean ± standard error of the mean (SEM). Statistical analyses were performed using the Mann-Whitney U test.

## RESULTS

### Characterization of antibody

To evaluate the specificity of our rabbit pAb to recombinant TREX1, we performed WB analyses. Western blotting of the recombinant *E. coli* protein used to immunize the rabbits detected a band for the recombinant V235fs TREX1 and a second band at ∼70 kDa (Fig. 1A). Mass spectroscopy identified this latter band as *E. coli* heat shock protein 70 (Hsp70), which has been reported to co-purify with some recombinant proteins.(23) Western blots for endogenous TREX1 in 293T cells also identified a cross-reacting protein at ∼70 kDa which was blocked by preincubation of the pAb to TREX1 with recombinant *E. coli* Hsp70 (Fig. 1B). In lysates from 293T cells transfected with YFP-tagged TREX1 (wild-type and V235fs), the rabbit anti-TREX1 Ab detects bands of the same mol wt as anti-YFP (not shown).

Two major bands at 37 and 32 kDa were detected in 293T cells (Fig. 1B) along with an unidentified light band at 27 kDa, intermittently seen on WBs, which may be a fragment of TREX1 as reported previously.(17) The band at 32 kDa corresponds to TREX1 transcript variant 4 (NM_033629.4), encoding isoform-B (NP_338599.1, 314 amino acids) of the wild-type protein (Fig. 1C). The band at 37 kDa likely represents TREX1 transcript variant 1 (NM_016381.5) encoding isoform-A (NP_057465.1, 369 amino acids), which is included in NCBI databases. Isoform-A retains the intron encoding a longer amino-terminus but is otherwise identical to isoform-B with respect to the amino acid sequence and localization (Fig. 1D). Although small amounts of the unspliced transcript encoding isoform-A have been found in human tissues (18), expression of the protein has not been reported in the literature. Both bands were observed however in human monocyte-derived macrophages from peripheral blood and four other human cell lines tested, namely HeLa, THP-1, U937, and Jurkat (not shown).

We next asked if the band at 37 kDa is indeed isoform-A of TREX1. The 37- and 32-kDa bands are endogenously expressed in HeLa cells and align with the recombinant wild-type TREX1 isoforms-A and B transiently expressed in 293T cells, respectively (Fig. 1E, lanes 1 and 2). The plasmid encoding the longer isoform-A also produces isoform-B (lane 2, Fig. 1E), likely from use of the downstream start site of transcript variant 4.

HeLa cells transfected with TREX1 siRNA (targeting sequences shared by both transcript variants) were then analyzed for changes in protein expression at 48 and 72 h. Although the levels of TREX1 isoform-B (32-kDa band) were found to be decreased at both time points, there was only a moderate decrease in the 37-kDa band at 72 h (Fig. 1E). As the 37-kDa band showed a greater decrease at 72 h, that time point was used for subsequent studies. Three independent experiments showed that at 72 h, compared to the negative control siRNA, the 32-kDa band was substantially reduced (18–35% of control), while the 37-kDa band was only moderately reduced (53–63% of control) by TREX1-specific siRNA (Supplementary Fig. 1A).

The presence of messenger RNA (mRNA) from both transcript variants was also studied in 293T, HeLa and EBV transformed B lymphocytes from normal controls and carriers of the RVCL frame-shift mutation (Supplementary Fig. 1B). Primers designed to anneal outside the retained intron in transcript variant 1 allowed differentiation of the two transcripts based on size. From RT-PCR of total mRNA from 293T cells, we observed two bands corresponding to transcripts 1 and 4 of TREX1. On the other hand, HeLa cells and the EBV transformed lymphocytes had one major band corresponding to transcript 4 and a faint band corresponding to transcript 1. The PCR, run in parallel on the mRNA from both cell lines, showed no product even though genomic DNA was able to amplify a band the same size as transcript 1. Thus, genomic DNA contamination was not responsible for the band attributed to transcript 1. To further verify the presence of transcript variant 1 in the cell lines examined, primers were designed to specifically amplify it. In all the cell lines examined, transcript variant 1 was detected by RT-PCR of mRNA but not PCR, which would amplify genomic DNA (Supplementary Fig. 1C). Thus, transcript variant 1 is expressed at the mRNA level and these results indicate that our Ab is detecting TREX1 isoform-A.

### Endogenous expression of TREX1 frame-shift mutants associated with RVCL

**Figure 2.**
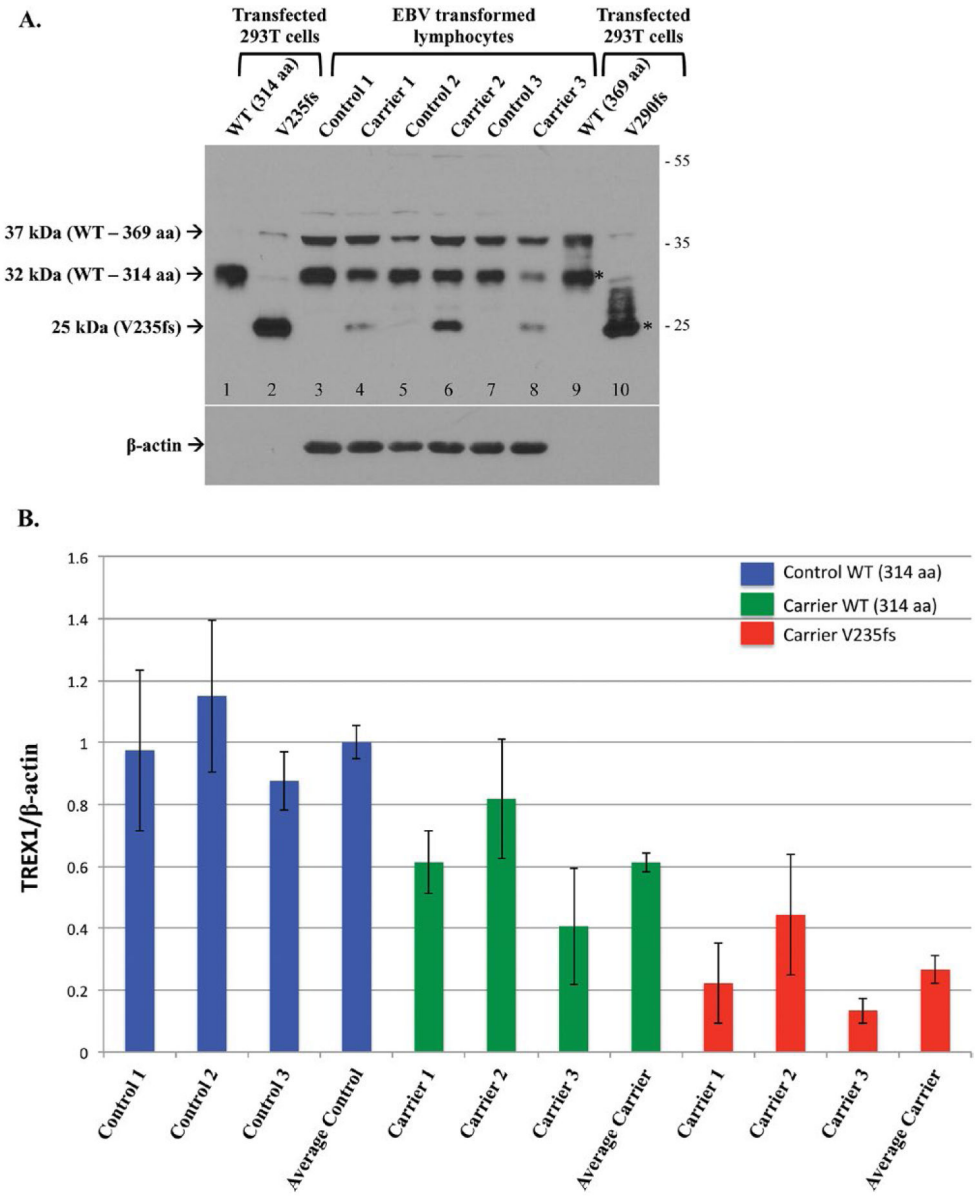
Endogenous expression of TREX1 in normal controls and heterozygous carriers of the V235fs mutant. **A.** Representative Western blot of transfected TREX1 in 293T cells (lanes 1–2, 9–10) and untransfected EBV transformed lymphocytes from normal controls and RVCL mutation carriers. The transfected protein is detected for both isoforms of wild-type (WT, lanes 1 and 9) and the frame-shift mutant of isoform-B (V235fs, lane 2). The frame-shift mutant of isoform-A (V290fs, lane 10) is not well seen. Both the wild-type and frame-shift mutant of isoform-A also produce the smaller isoform-B (*). Endogenous WT TREX1 is detected in all EBV transformed lymphocytes. The 37 kDa band represents TREX1 isoform-A (lanes 3–8). The V235fs mutant is detected in cell lines derived from heterozygous carriers of the mutation (lanes 4, 6, and 8) but not in normal controls (lanes 3, 5, and 7). Endogenous expression of the V290fs mutant is not detected. Membrane was stripped and reprobed with anti-β-actin as a loading control. **B.** The expression of TREX1 protein was normalized to a β-actin loading control. For each group, Control WT, Carrier WT and Carrier V235fs, data are shown for the 3 individuals analyzed followed by the average for that group. TREX1 levels are given relative to the average of WT in the 3 normal controls (Average Control), which was arbitrarily set to 1.0. Data are presented as mean ± SD and represent 8 independent samples.

Using EBV transformed B-lymphocytes from normal controls and carriers heterozygous for the V235fs mutant, the endogenous expression of TREX1 was evaluated by WB (Fig. 2). 293T cells transfected with both isoforms of wild-type TREX1 and the frame-shift mutants were included as positive controls (Fig. 2A, lanes 1–2 and 9–10). The proteins from transcript variant 4 (corresponding to isoform-B) were detected at their expected molecular weight (32 kDa and 25 kDa for wild-type and V235fs mutant, respectively; lanes 1 and 2). As noted above, the wild-type isoform-A was detected at 37 kDa but the transfected cells also produced similar amounts of isoform-B (32 kDa; lane 9). The RVCL mutation in isoform-A, V290fs, should be detected at ∼30 kDa. However, the transfected cell lysates produced a smear, probably due to degradation, and a 25-kDa band corresponding to the V235fs mutant (lane 10).

Endogenous levels of TREX1 were observed in the EBV transformed B lymphocytes from normal controls and heterozygous carriers of the RVCL mutation (Fig. 2A, lanes 3–8). For each subject, 1 × 10^6^ cell equivalents were loaded to detect the protein (with a 1-min exposure), though transfected protein was detected in ∼3 × 10^5^ cell equivalents. In contrast, β-actin levels were easily detected (with a 2 s exposure) for each subject but not for the transfected cell lysates, which indicates that TREX1 is expressed endogenously at low levels. Wild-type TREX1 was observed in all, regardless of genotype. However, the amount of wild-type isoform-B in carriers of the frame-shift mutation was about 60% (range 41–82%; 50% expected) of that present in normal controls (Fig. 2B). The levels of the 37-kDa band, likely representing TREX1 isoform-A, did not show the same variability with respect to genotype. In addition, the 37-kDa band did not have a consistent pattern with respect to the 32-kDa band; sometimes both were detected in equal amounts while at other times one or the other was more highly expressed.

As expected, the endogenous V235fs mutant was observed only in carriers with the RVCL mutation (Fig. 2A, lanes 4, 6, and 8). The expression of the V235fs mutant was ∼27% (range 13–44%) of wild-type TREX1 isoform-B in normal controls (Fig. 2B), less than the 50% expected in heterozygous carriers. The V290fs, which was poorly detected even in transfected cell lysates, was not detected in any of the carriers. This may be because the V290fs is not stable when expressed (similar to the transfected protein) or is expressed below the level of detection.

### Optimization of rabbit pAb to TREX1 for staining in human brain tissue

We next investigated the utility of the rabbit pAb for staining of tissue sections from human brain. Although the serum from the final bleed was satisfactorily employed for WB, it resulted in a high background when staining tissue. However, a two-step process of purifying the IgG fraction from the saturated ammonium sulfate precipitation over an antigen-affinity column yielded an Ab preparation with minimal background on tissue staining. This Ab was used for all immunofluorescence and immunohistochemistry studies.

**Figure 3.**
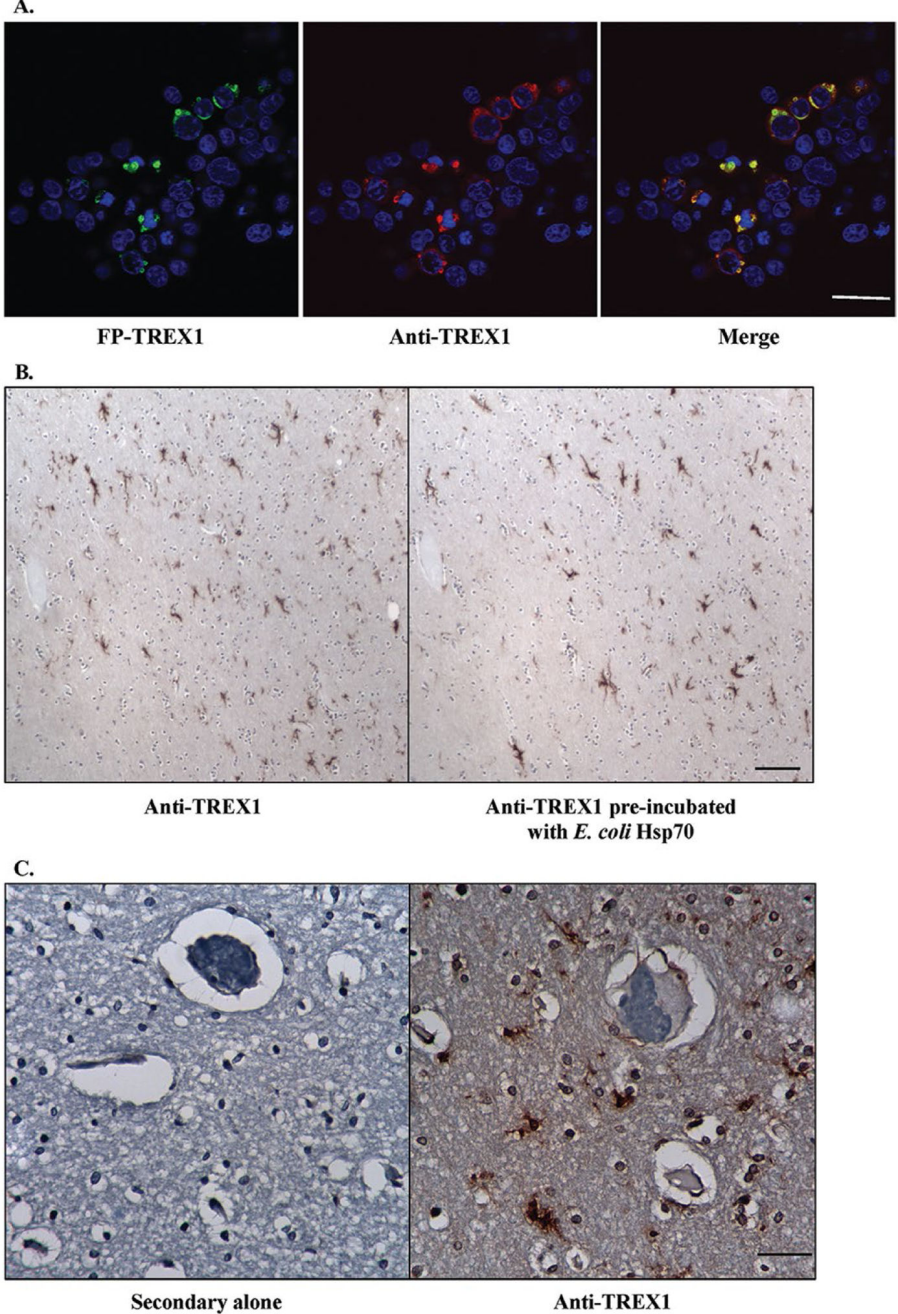
Specificity of rabbit pAb to TREX1. **A.** Immunofluorescent staining of 293T cells transfected with fluorescent protein-tagged wild-type TREX1 (FP-TREX, green) using the rabbit pAb to TREX1 (red) shows that the signals overlap (yellow). Scale bar represents 28 μm. **B.** Immunohistochemical staining of human brain tissue with rabbit pAb to TREX1 alone (left panel) and after pre-incubation of the Ab with *E. coli* heat shock protein 70 (Hsp70, right panel). Scale bar represents 100 μm. **C.** Immunohistochemical staining of human brain tissue with secondary Ab alone (left panel) or rabbit pAb to TREX1 (right panel). Scale bar represents 40 μm.

In 293T cells transfected with FP-tagged TREX1, the Ab only stained transfected cells but not untransfected cells (Fig. 3A). In order to verify that the staining was not due to the cross-reacting band observed at ∼70 kDa on WB, we stained sections with the Ab alone and Ab pre-incubated with *E. coli* Hsp70 (Fig. 3B). Equivalent staining was observed for both indicating that the Ab preparation used was specific for TREX1. .No staining was observed in tissue sections stained with secondary Ab alone as a negative control (Fig. 3C). These data demonstrate the specificity of the Ab in staining for TREX1.

### TREX1 expression in human brain tissue is restricted to a subset of microglia and macrophages

**Figure 4.**
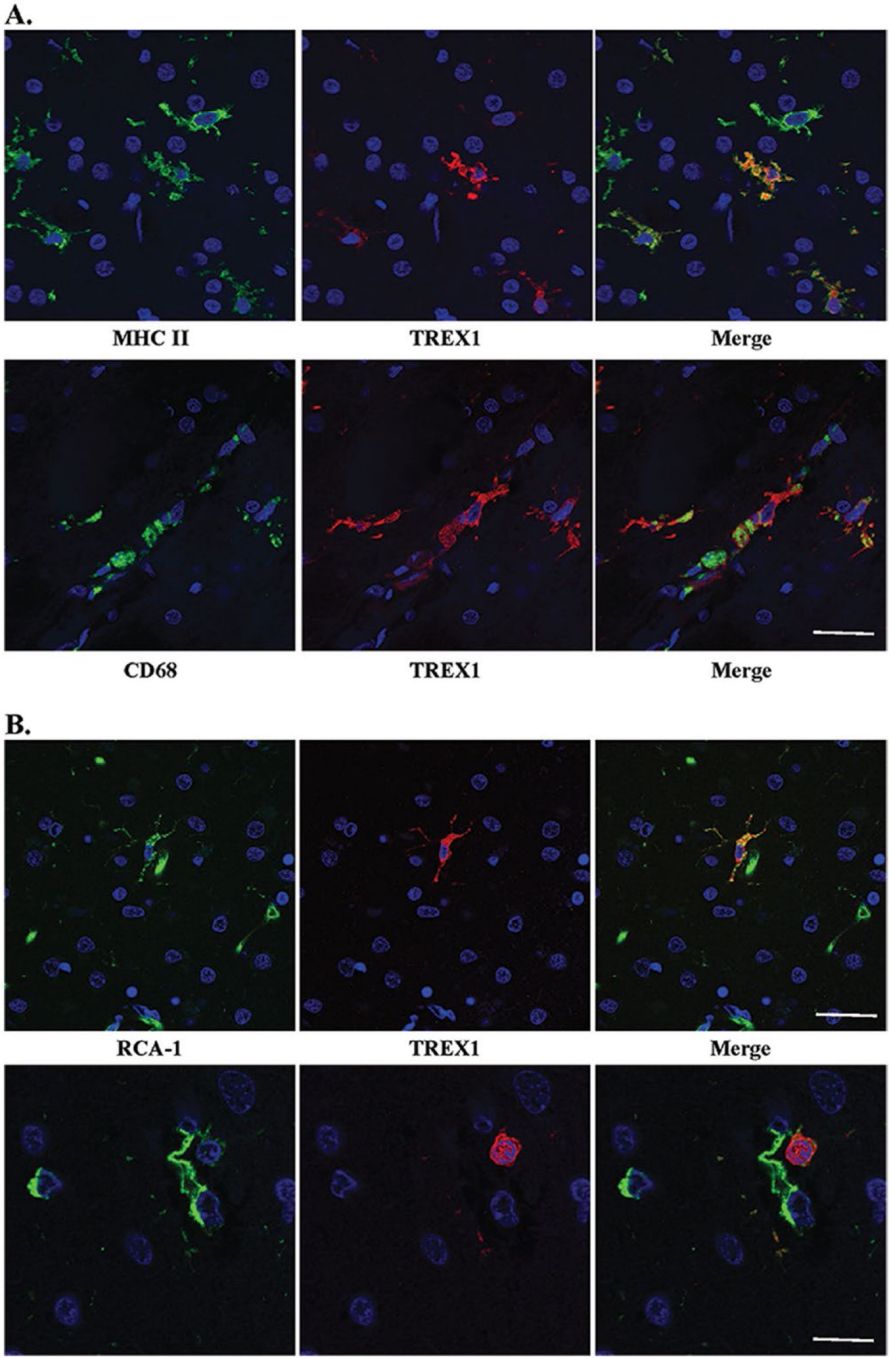
TREX1 positive cells are microglia and/or macrophages. **A.** Dual staining of frozen tissue from a case of RVCL with anti-MHC II (top panel, green) and anti-CD68 (bottom panel, green), microglial markers, and anti-TREX1 (red). Nuclei are counterstained with TO-PRO-3 (blue). Scale bar represents 28 μm. **B.** Two examples (upper and lower panels) of dual staining of formalin-fixed, paraffin-embedded human brain tissue from a normal control with RCA-1 (left panel, green), a microglial/macrophage and endothelial cell marker, and anti-TREX1 (red). Nuclei are counterstained with TO-PRO-3 (blue). Some cells stained with RCA-1 but not TREX1 are endothelial cells based on their morphology. Scale bars represent 28 μm on top panel and 14 μm on bottom panel.

We next asked which cells express TREX1 in human brain. Although we had hypothesized that TREX1 would be expressed by endothelial cells given the vasculopathy observed in RVCL, immunohistochemical staining demonstrated that the protein was in glial cells. Frozen sections from autopsy material obtained from a patient with RVCL were used for immunofluorescent dual-staining with several glial markers. Based on the morphology of the TREX1^+^ cells, astrocytes or microglia were the most likely candidates. Dual staining with GFAP, an astrocyte marker, demonstrated no overlap with the cells stained for TREX1 (Supplementary Fig. 2). CD68 and MHC II, markers for microglia and macrophages, colocalized with all TREX1^+^ cells (Fig. 4A). In almost all cases, the TREX1^+^ cells were likely to be microglia as they had the typical ramified morphology and were found within the parenchyma. While all TREX1^+^ cells were identified by these markers, not all microglia and macrophages stained positive for TREX1.

To determine if TREX1^+^ cells are microglia in brain tissue from normal controls, we performed immunofluorescent dual-staining on FFPE autopsy tissue. Although CD68 and MHC II did not stain well on FFPE tissue, RCA-1 did. RCA-1 is a lectin that serves as a marker for microglial/macrophages and endothelial cells, which can be differentiated based on morphology. Similar to the findings observed in frozen sections, the TREX1^+^ cells were predominantly microglia (Fig. 4B), but not all microglia were TREX1^+^. Some TREX1^+^ cells around the vasculature had a rounded morphology, which may be representative of perivascular macrophages (Fig. 4B, bottom panel). Thus, TREX1 is a marker for a subset of microglia and perivascular macrophages in human brain tissue.

### Expression of TREX1 in human brain tissue from normal controls

To determine the distribution of TREX1^+^ cells and the proportion of microglia they represent in the normal human brain, we performed immunohistochemical staining on serial sections using TREX1 and Iba1, a marker for microglia and macrophages. Using the Abs in parallel allowed us to control for variability in staining due to fixation and the age of the sections as well as control for differences in cellular density. Absolute cell counts are provided in Supplementary Fig. 3.

**Figure 5.**
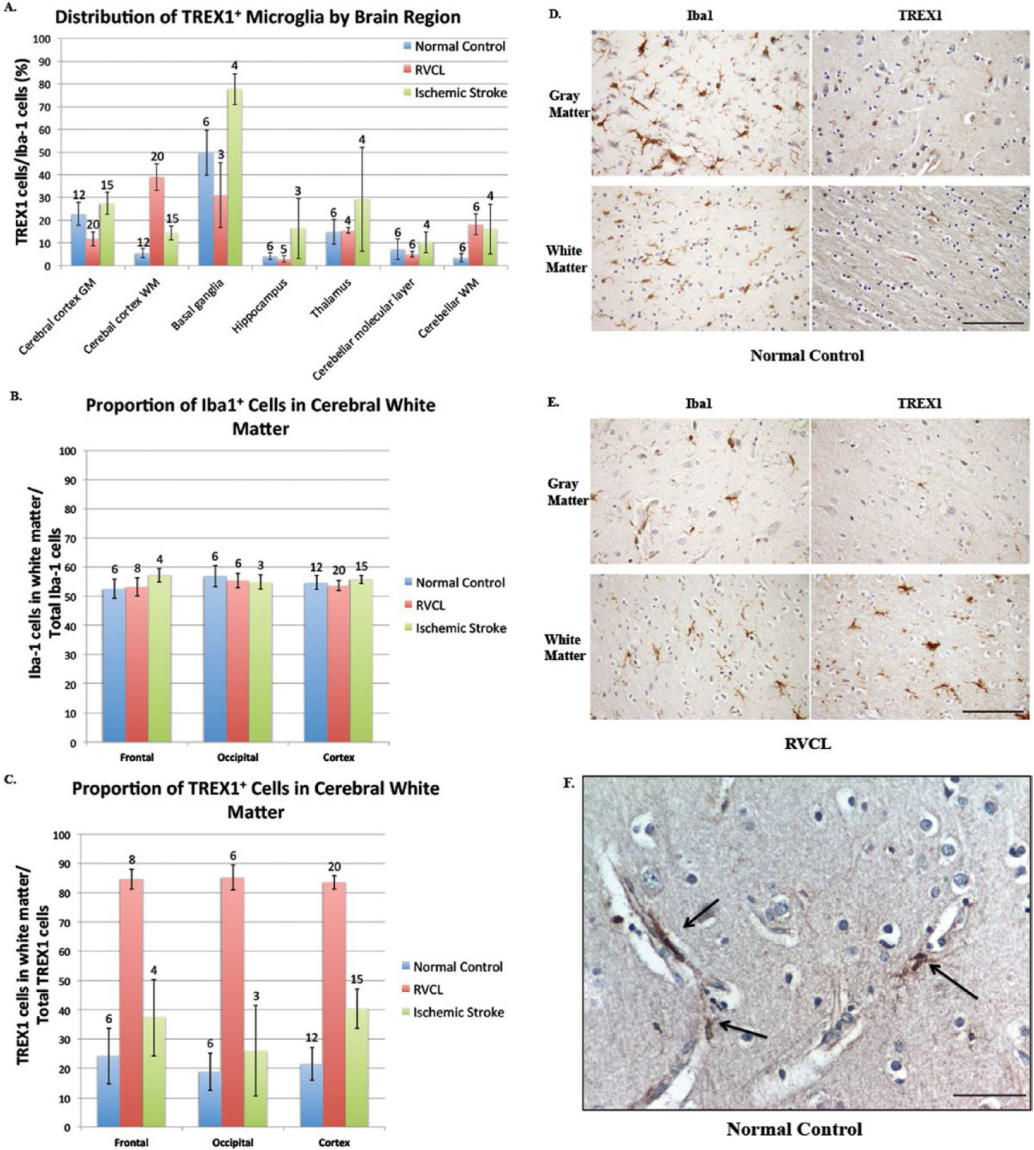
Distribution of TREX1 expressing cells. **A.** Distribution of TREX1^+^ microglia by brain regions in normal controls and cases of RVCL and ischemic stroke. The proportion of TREX1 cells is shown relative to the mean number of microglia (Iba1 stained cells) for specific areas of the brain in normal controls (blue bars) and cases with RVCL (red bars) or ischemic stroke (green bars). Sections quantified in RVCL and ischemic stroke were taken from undamaged tissue. The number of tissue sections included for each observation is noted above the appropriate column. White matter (WM); Gray matter (GM). Data are presented as mean ± SEM. **B-C.** Distribution of TREX1 and Iba1 staining in the cerebral cortex of normal controls and cases of RVCL and ischemic stroke. The proportion of Iba1 (E) or TREX1 (F) cells located in the white matter for the frontal lobe, occipital lobe and the cerebral cortex in normal controls (blue bars) and cases with RVCL (red bars) or ischemic stroke (green bars). The values represent the mean among each group for cell counts in the white matter divided by the total cell counts in the white and gray matter for the specified region. Sections quantified in RVCL and ischemic stroke were taken from undamaged tissue. The cerebral cortex includes sections from the frontal, parietal, temporal and occipital lobes. The number of tissue sections included for each observation is noted above each column. Data are presented as mean ± SEM. **D-E.** Immunohistochemical staining of a normal control and subject with RVCL with Iba1 (left panels) and TREX1 (right panels) in the gray (top panels) and white matter (bottom panels). For RVCL, undamaged brain tissue is shown. Staining for Iba1 and TREX1 is developed with DAB (brown). Nuclei are counterstained with hematoxylin (blue). Scale bars represent 100 μm. **F.** Representative immunohistochemical staining of normal control gray matter with TREX1 microglia (DAB, brown) in association with microvasculature (arrows). Nuclei are counterstained with hematoxylin (blue). Scale bar represents 40 μm.

The expression of TREX1 varied considerably among the brain regions examined (Fig. 5A). The percent of TREX1 expressing microglia (mean of TREX1^+^ cell count/Iba1^+^ cell count within the specified brain region for all individuals in the group) was highest in the basal ganglia (49.8 ± 9.9% of microglia) followed by the gray matter of the cerebral cortex. Unlike the gray matter, fewer TREX1^+^ microglia were identified in the white matter of the cerebral cortex (22.8 ± 5.1% of microglia in gray matter vs. 5.5 ± 1.9% of microglia in white matter, p ≤ 0.01). Although the microglia were equally distributed in the gray and white matter of the cerebral cortex (Iba1 stain, 54.8 ± 2.4% in white matter), TREX1^+^ microglia were observed less frequently in the white matter (TREX1 stain, 21.6 ± 5.5% in white matter; Fig. 5B-D). As such, the difference in the distribution of TREX1^+^ cells between the gray and white matter is not due to a difference in the distribution of microglia overall. In addition, while the Iba1^+^ cells were uniformly spread throughout the parenchyma of the gray and white matter, TREX1^+^ cells were not. The TREX1+ microglia in the cerebral cortex were often closely associated with the microvasculature, particularly in the gray matter (Fig. 5F). These TREX1^+^ microglia were either flush against the vessel or had foot processes extending to the vessel perimeter. Dual staining with anti-TREX1 and antialpha smooth muscle actin, a marker for vascular smooth muscle cells and pericytes, showed no co-localization (not shown).

In the hippocampus and the deep gray matter of the thalamus and basal ganglia, the TREX1^+^ cells were scattered throughout the parenchyma (Supplementary Fig. 4). No differences were observed in the distribution of TREX1^+^ microglia between the hippocampus, subiculum and parahippocampus. In the basal ganglia, the TREX1^+^ microglia were noted in both the white matter tracts (pencil fibers of Wilson) as well as the surrounding gray matter. In the cerebellum, the TREX1^+^ cells were present in the molecular layer and white matter. TREX1 expressing microglia were associated with the dentate nucleus in the cerebellum of all normal controls (n=5, dentate nucleus not included on section from 1 subject). Except for the cerebral cortical gray matter and basal ganglia, the percent of microglia expressing TREX1 was less than 15% in the regions quantified.

### Expression of TREX1 in undamaged brain tissue from subjects with RVCL

We next studied the expression of TREX1 in the brain from cases of RVCL in histologically normal appearing tissue. Although there was variability in the percent of TREX1 expressing cells in the brain regions examined, which was also noted in normal controls, there were differences between the two groups (Fig. 5). The most striking distinction was the increased expression of TREX1 in the white matter of the cerebral cortex from brain tissue of RVCL cases (5.5 ±1.9% of Iba1^+^ microglia in normal controls vs. 38.9 ± 5.8% in RVCL, p ≤ 0.01; Fig. 5A-E). In addition, TREX1 expressing cells in the gray matter of RVCL cases were decreased compared to normal controls (22.7 ± 5.1% in controls vs. 11.8 ± 3.1% in RVCL, p ≤ 0.02). Although the proportion of Iba1^+^ microglia in the white matter was similar to that of normal controls (54.8 ± 2.4% in controls vs. 53.6 ± 1.7% in RVCL, p = n.s.; Fig. 5B), there was a marked increase in the proportion of TREX1^+^ cells in the white matter (21.6 ± 5.5% in controls vs. 83.5 ± 2.3% in RVCL, p ≤ 0.01; Fig. 5C). Thus, compared to normal controls, the TREX1^+^ cells are increased in the white matter and decreased in the gray matter of RVCL cases, even though the distribution of microglia between the gray and white matter is similar in both groups. The increased expression of TREX1 in the white matter is also observed in the cerebellum of RVCL cases compared with normal controls (Fig. 5A).

Another difference between RVCL and normal control brain tissue was the distribution of TREX1^+^ cells within the white matter. In RVCL (within histologically normal white matter), there were areas without TREX1^+^ cells while other regions on the same tissue section had clusters of TREX1^+^ cells. This is in contrast to normal controls where the TREX1 expressing cells were more uniformly distributed.

With the exception of the white matter regions, the expression of TREX1 was reduced or similar to that seen in normal controls in other brain regions (Fig. 5A, Supplementary Fig. 3). Within the thalamus, the TREX1^+^ microglia could be observed scattered in both the gray and white matter. Similarly, in the basal ganglia the TREX1^+^ cells were located within the white matter tracts (pencil fibers of Wilson) and in the adjacent gray matter. In some cases, the pencil fibers of Wilson showed TREX1^+^ cells with a rounded morphology, similar to macrophages, even though no lesions were observed in the adjacent tissue on the section. TREX1^+^ cells were rarely found in the hippocampus. In the cerebellum, TREX1 was primarily noted in the white matter while the molecular layer had occasional scattered TREX1^+^ microglia. The dentate nucleus was only included in a tissue section from 1 case with RVCL making it difficult to compare to the findings in normal controls. Except for the white matter and basal ganglia, TREX1^+^ microglia represent less than ∼15% of all microglia in other regions, similar to our observations in normal controls.

### Expression of TREX1 in ischemic lesions

**Figure 6.**
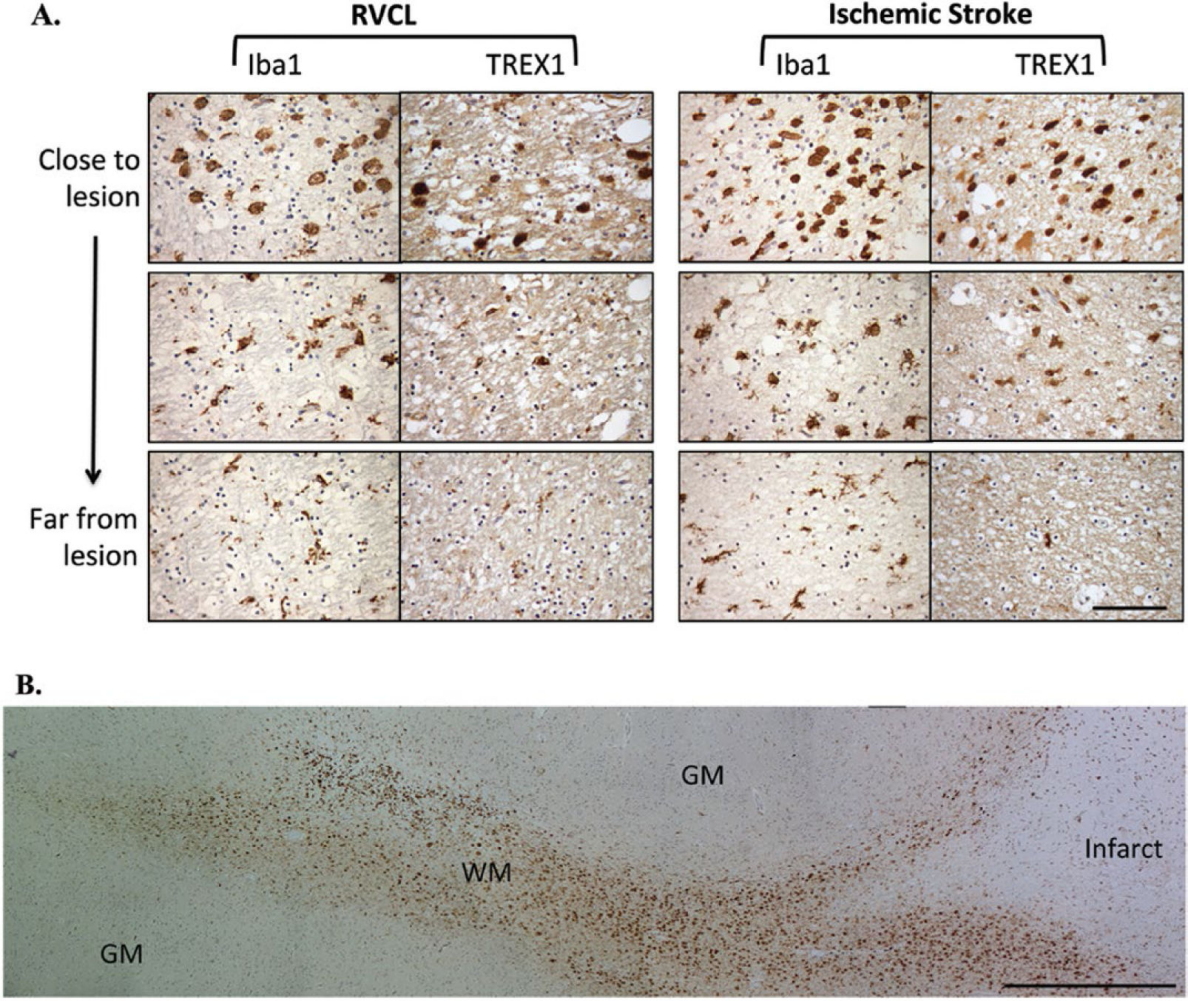
Immunohistochemical staining in white matter lesions with anti-TREX1. **A.** Staining for Iba1 and TREX1 in white matter lesions from cases with RVCL (left) or ischemic stroke (right). Representative images from chronic lesions are shown (400x magnification) with each successive image taken from two high-powered fields away. Staining for Iba1 and TREX1 is developed with DAB (brown). Nuclei are counterstained with hematoxylin (blue). Scale bar represents 100 μm. **B.** Panoramic view of the morphological changes in TREX1^+^ cells around a chronic lesion in ischemic stroke (right side) and their tracking in the adjacent white matter (WM) with relative absence of TREX1^+^ cells in the gray matter (GM). Similar findings are noted in RVCL lesions. Scale bar represents 1 mm.

We next evaluated the expression of TREX1 in the white matter lesions of RVCL cases. The neuropathological findings observed in RVCL consist of scattered areas of ischemic necrosis in the white matter associated with wall thickening and stenosis of the small blood vessels. (26,12) Given that RVCL results in occlusion or loss of small blood vessels, we also compared our findings to cases of ischemic stroke as that is also a result of disruption in blood flow. In ischemic lesions, there was a distinct pattern in most cases (11 sections from five subjects with RVCL and 13 sections from four subjects with stroke). In close proximity to large necrotic areas, often with calcifications, few TREX1^+^ cells were present. In many cases, only an empty cavity surrounded by a variable necrotic zone remained. Bordering the lesions, a subset of the round, foamy cells expressed TREX1. Dual staining with RCA-1 and TREX1 demonstrated co-localization (Supplementary Fig. 5). The cell morphology in these cases is similar to that of activated, ameboid microglia or infiltrating macrophages, cell types that cannot be distinguished in human brain tissue sections with current markers. Moving away from the lesion, the TREX1^+^ cells gradually change their morphology to a more ramified appearing microglia (Fig. 6A, bottom panel). In ischemic stroke cases, these TREX1^+^ cells are observed in distinct tracts, usually within the adjoining white matter (Fig. 6B). Other lesions had none or faintly stained TREX1^+^ cells (five sections from four subjects with RVCL and two sections from one subject with stroke). The staining was unchanged after pre-incubation of the Ab with Hsp70 (not shown).

To determine if the differences noted between normal controls and RVCL were applicable to ischemic injury in general or were specific to RVCL, we also quantified the expression of TREX1 in sections consisting of undamaged brain tissue from cases with ischemic stroke. All of the groups studied demonstrated variability between different brain regions with respect to the percent of microglia expressing TREX1 (Fig. 5A). In general, there was a greater proportion of TREX1^+^ microglia in all brain regions with stroke compared to normal controls and most regions examined in RVCL cases. This increase is likely in response to the ischemic injury, as it was not observed in other neuropathologies screened (multiple sclerosis, radiation necrosis and brain tumors; not shown). Only in the white matter of the cerebral cortex did RVCL cases have the highest expression of TREX1 (38.9 ± 5.8% of Iba1^+^ microglia for RVCL vs. 14.5 ± 3.1% for ischemic stroke, p ≤ 0.01). Similar to normal controls and RVCL cases, microglia were evenly distributed between the white and gray matter in ischemic stroke (55.0 ± 1.3% of Iba1^+^ cells in white matter, Fig. 5B). In contrast, the proportion of TREX1^+^ cells in the white matter was increased compared to normal controls but not as highly as in RVCL (40.4 ± 6.8% in ischemic stroke, Fig. 5C).

More TREX1^+^ microglia were observed in ischemic stroke compared with normal controls for all brain regions. In the cerebellum, an association of TREX1^+^ cells with the dentate nucleus was observed in all stroke cases (n=4), similar to our findings in normal controls. However, despite the increased expression of TREX1 in ischemic stroke, these cells represent less than 30% of microglia in all brain regions examined, with the exception of the basal ganglia (77.7 ± 6.8% of TREX1^+^ microglia).

## DISCUSSION

We present here the development and characterization of a specific rabbit pAb to TREX1 that detects this intracellular protein by WB and tissue staining. Although endogenous TREX1 protein from cell lysates has been observed by WB,(8,25) our study is the only one to detect TREX1 isoform-A (37 kDa) and we confirmed the endogenous expression of the frame-shift mutants associated with RVCL. We also demonstrate the expression of the protein in human brain tissue for the first time. Further, dual staining demonstrated that TREX1 is exclusively expressed by a subset of microglia and macrophages at our level of detection.

Microglia are the tissue resident macrophages of the central nervous system (CNS) where they serve multiple functions.(2,21) During development they prune extra synapses and facilitate the establishment of the vascular network in the CNS.(1,28) They also perform active surveillance of the surrounding tissue to maintain homeostasis and respond rapidly to perceived insults such as injury or pathogens. Although microglia have been implicated in a number of neurodegenerative diseases including Alzheimer’s disease, Parkinson’s disease, ischemic stroke and multiple sclerosis, our understanding of their primary role in the brain is incomplete. Initially thought to represent a homogenous population, recently the existence of microglial subsets with specialized functions has been proposed.(7) Our results, which show the expression of TREX1 is limited to a subset of microglia, strongly support this view. The variation in expression of TREX1 by brain region also suggests a specialized physiologic role.

The association of TREX1^+^ microglia with the microvasculature, particularly notable in normal control gray matter, suggests a role for these cells in the development and/or maintenance of vessels within the CNS. While we had initially hypothesized that TREX1 would be expressed in endothelial cells, as RVCL is a systemic vasculopathy, the observation that the protein is most highly expressed in microglia suggests an alternative that should be considered for any work pursued in the future. Furthermore, as microglia are limited to the CNS, this may explain, in part, the prominent vasculopathy affecting the white matter and retina in RVCL. Less damage in other organs from this systemic small vessel disease may be attributable to differences among tissue resident macrophages, more efficient repair processes or greater biological reserve. Future studies on tissues of other organs from subjects with RVCL are needed to determine where TREX1 is expressed and in which cell types.

Our studies on TREX1 expression in human brain tissue also demonstrate significant increases in the undamaged cerebral white matter of cases with RVCL compared to normal controls and ischemic stroke. The increase of TREX1^+^ microglia in all regions of the brain in ischemic stroke compared to normal controls suggests that this is a general response to ischemia. As such, the increased expression of TREX1 in the undamaged white matter of RVCL may be indicative of wide-spread ongoing injury that is undetectable histologically. The clusters of TREX1^+^ microglia in the white matter of RVCL may correlate with this low-level injury (and possibly exacerbate it). In addition, the sparing of the gray matter in RVCL may explain why TREX1^+^ microglia are less frequent there in comparison to ischemic stroke (where gray matter can be affected). However, it does not account for why TREX1^+^ microglia are decreased in the cerebral gray matter of RVCL compared to normal controls.

Another possibility is that the expression of TREX1 in RVCL cases is not the result of tissue injury but rather causes the damage. Regions of the brain that are able to limit the expression of the mutant TREX1 are therefore spared; though how this is regulated remains unexplained. In order to differentiate between mutant TREX1 expression causing the white matter damage in RVCL or being increased as result of injury, an animal model that develops the clinical manifestations would be valuable.

A role for TREX1 in ischemia is further supported by our findings in the lesions of RVCL and ischemic stroke. In most lesions, increased expression of TREX1 was observed in ameboid microglia and/or infiltrating macrophages. This increase of TREX1^+^ microglia may be due to migration toward the infarct to assist in clearance. These findings are consistent with the known response of microglial to injury wherein microglia transition to an ameboid morphology and migrate toward sites of sterile injury.(9,27) We hypothesize that TREX1 assists in phagocytosis and clearance of necrotic debris in both macrophages and microglia. Although TREX1 degrades cytosolic DNA in the cytoplasm, it has not been detected in lysosomal compartments.(34) However, it is possible that, in the setting of large necrotic lesions, TREX1 becomes involved in degrading DNA waste and that defects in clearance of nucleic acids may exacerbate ischemic injury.

Alternatively, TREX1 may serve another function unrelated to its exonuclease activity, such as limiting the inflammatory activation of phagocytes.(22,25) It is possible that RVCL mutants impair this function, which may worsen an ischemic insult.

One limitation of our study is that the samples from patients with RVCL were predominantly from those carrying the V235fs as that is the mutation found in the families cared for at our institution. However, this mutation is the one most commonly found across the globe in unrelated individuals, being present in approximately 50% of the families described to date [26 and M.K. Liszewski and J. P. Atkinson, unpublished]. Thus, it is possible that the individual mutations may demonstrate differences in the expression of TREX1. However, that is unlikely as the clinical manifestations of RVCL are similar across the various mutations discovered to date (4,32) and the one case carrying the T249fs mutation demonstrated findings that were consistent with what was observed with the V235fs mutation. Additional studies including brain tissue from subjects with the other RVCL-associated mutations will be needed to determine if there is any variability. Another limitation of our study is that we are only able to observe TREX1 if it was expressed at levels detectable by tissue staining. In Western blots we have been able to detect endogenous TREX1, albeit in large samples, in various different cell types. As such, it is probable that TREX1 is expressed in additional cell types. Regardless, the detection of TREX1 in only a subset of microglia and perivascular macrophages suggests that these cell types are of particular relevance in future studies of RVCL. Additional studies are also needed to determine if the expression of TREX1 in the microglia plays a role in other diseases associated with mutations in TREX1, such as AGS, SLE and FCL, or if there are differences in the expression of TREX1 by organs and/or cell types.

In summary, we generated a highly specific rabbit pAb that is able to detect endogenous levels of human TREX1 by WB and tissue staining. With this Ab we localized TREX1 to a subset of microglia in human brain tissue. Our staining demonstrates an association between these TREX1^+^ microglia and the microvasculature in normal, undamaged tissue. The function of these cells still needs to be determined but it is possible that the TREX1^+^ microglia are a specialized subset involved in vessel homeostasis and/or the response to ischemic injury. The increase in TREX1 expression in ischemic stroke strongly indicates that it plays a role in the handling of necrotic debris. Further studies clarifying this exact relationship of TREX1^+^ microglia with the microvasculature and with ischemic lesions should provide insight into how a functional but mislocalized exonuclease results in the clinical manifestations of RVCL.(8,26)

## Supplementary Material

SupFig1 **Figure S1.** Expression of TREX1 protein isoforms and transcript variants. A. The level of TREX1 protein was normalized to a β-actin loading control. The level of TREX1 was expressed relative to the negative control (NC1) which was arbitrarily set to 1.0. Light and dark blue bars represent the 37- and 32-kDa bands, respectively. Data, presented as mean ± SD, are the result of three independent samples. B. Reverse-transcriptase PCR (RT-PCR) of DNase I treated RNA from 293T (lane 1), HeLa (lane 2) cells, and EBV-transformed lymphocytes from carriers of the V235fs mutation (lanes 3 and 5) and controls (lanes 4 and 6). PCR of DNase I treated RNA serves as negative controls for the RT-PCR (lanes 7–12). PCR of genomic DNA (gDNA) from 293T (lane 13) and HeLa (lane 14) serve as positive controls for the PCR reaction. Transcript variants 1 and 4 produce ∼600 and 270 base pair products, respectively, with primers that align to regions shared between both transcripts and surrounding the retained intron in transcript variant 1. C. Same as B with primers designed to detect transcript variant 1 only as a ∼300 base pair product.

SupFig2 **Figure S2.** TREX1 expressing cells are not astrocytes. Dual staining of formalin-fixed, paraffin-embedded human brain tissue from a case of RVCL with anti-glial fibrillary acidic protein (GFAP, green), a marker for astrocytes, and anti-TREX1 (red). Scale bar represents 21 μm.

SupFig3 **Figure S3.** Absolute cell counts for Iba1 and TREX1 by brain region in normal controls and cases of RVCL and ischemic stroke. The cell counts for Iba1 (A) and TREX1 (B) for specific areas of the brain in normal controls (blue bars), cases with RVCL (red bars) and cases with ischemic stroke (green bars). Sections quantified in RVCL and ischemic stroke were taken from undamaged tissue. The number of tissue sections included for each observation is noted above each column. White matter (WM); Gray matter (GM). Data are presented as mean ± SEM.

SupFig4 **Figure S4.** Representative immunohistochemical staining for TREX1 (brown) in normal controls (left panel) and undamaged tissue in RVCL cases (right panel) in specified brain regions. Nuclei are counterstained with hematoxylin (blue). Scale bar represents 100 μm.

SupFig5 **Figure S5.** TREX1 cells are ameboid microglia or infiltrating peripheral macrophages along the edges of ischemic lesions. Dual staining of formalin-fixed, paraffin-embedded human brain tissue from a case of ischemic stroke with RCA-1 (left panel, green), a microglial/macrophage and endothelial cell marker, and anti-TREX1 (red). Nuclei are counterstained with TO-PRO-3 (blue). Scale bar represents 28 μm.

Supp Table Table S1. Summary of the tissue sections analyzed.

## Figures and Tables

**Table 1.** Clinical and pathological features of cases and controls

	Normal Controls	RVCL	Ischemic Stroke
	
*n*	6	5^*^	4
Age at death (years)	60.3 ± 5.2 (range 52–66)	56.0 ± 4.2 (range 50–60)	53.3 ± 2.5 (range 52–57)
Female gender	3 (50%)	2 (40%)	2 (50%)
Brain weight(g)	1251 ± 107	1128 ± 181^†^	1277 ± 93
Post-mortem interval (h)	37.2 ± 32.6	6.1 ± 2.5^†^	20.5 ± 13.8

RVCL, Retinal Vasculopathy with Cerebral Encephalopathy.

* Tissue from all brain sections unavailable for one subject.

† Data available on four of the five RVCL cases.
