# Supplementary figures and images for: TREX1 is expressed by microglia in normal human brain and increases in regions affected by ischemia
Source: Brain Pathol. 2018 Oct 10;28(6):806–21. doi: 10.1111/bpa.12626 (PMC6404532; doi:10.1111/bpa.12626)

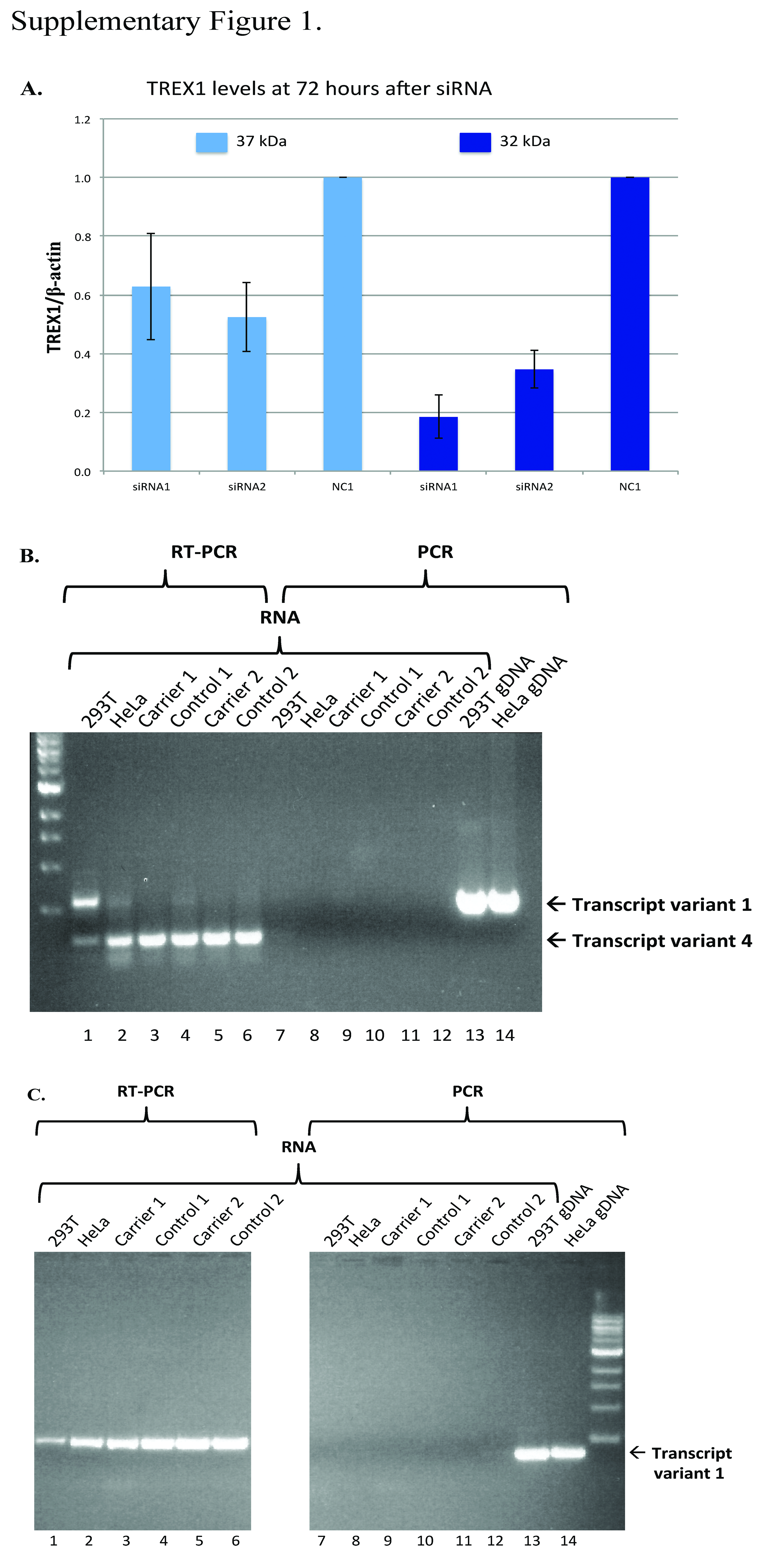

Supplement: Supplementary file 1 — Figure S1. Expression of TREX1 protein isoforms and transcript variants. A. The level of TREX1 protein was normalized to a β‐actin loading control. The level of TREX1 was expressed relative to the negative control (NC1) which was arbitrarily set to 1.0. Light and dark blue bars represent the 37‐ and 32‐kDa bands, respectively. Data, presented as mean ± SD, are the result of three independent samples. B. Reverse‐transcriptase PCR (RT‐PCR) of DNase I treated RNA from 293T (lane 1), HeLa (lane 2) cells, and EBV‐transformed lymphocytes from carriers of the V235fs mutation (lanes 3 and 5) and controls (lanes 4 and 6). PCR of DNase I treated RNA serves as negative controls for the RT‐PCR (lanes 7–12). PCR of genomic DNA (gDNA) from 293T (lane 13) and HeLa (lane 14) serve as positive controls for the PCR reaction. Transcript variants 1 and 4 produce ~600 and 270 base pair products, respectively, with primers that align to regions shared between both transcripts and surrounding the retained intron in transcript variant 1. C. Same as B with primers designed to detect transcript variant 1 only as a ~300 base pair product. [file BPA-28-806-s005.tif]

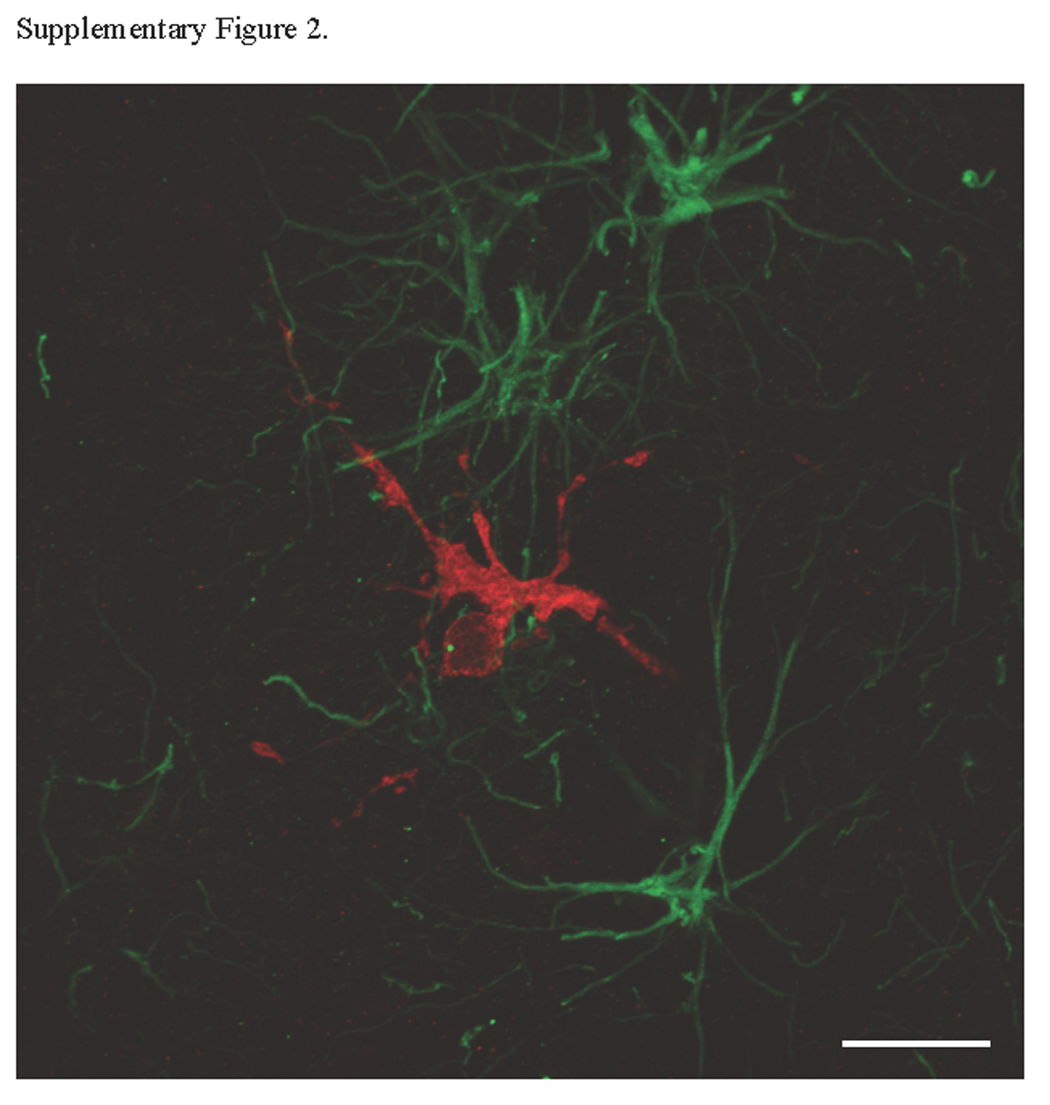

Supplement: Supplementary file 2 — Figure S2. TREX1 expressing cells are not astrocytes. Dual staining of formalin‐fixed, paraffin‐embedded human brain tissue from a case of RVCL with anti‐glial fibrillary acidic protein (GFAP, green), a marker for astrocytes, and anti‐TREX1 (red). Scale bar represents 21 μm. [file BPA-28-806-s004.tiff]

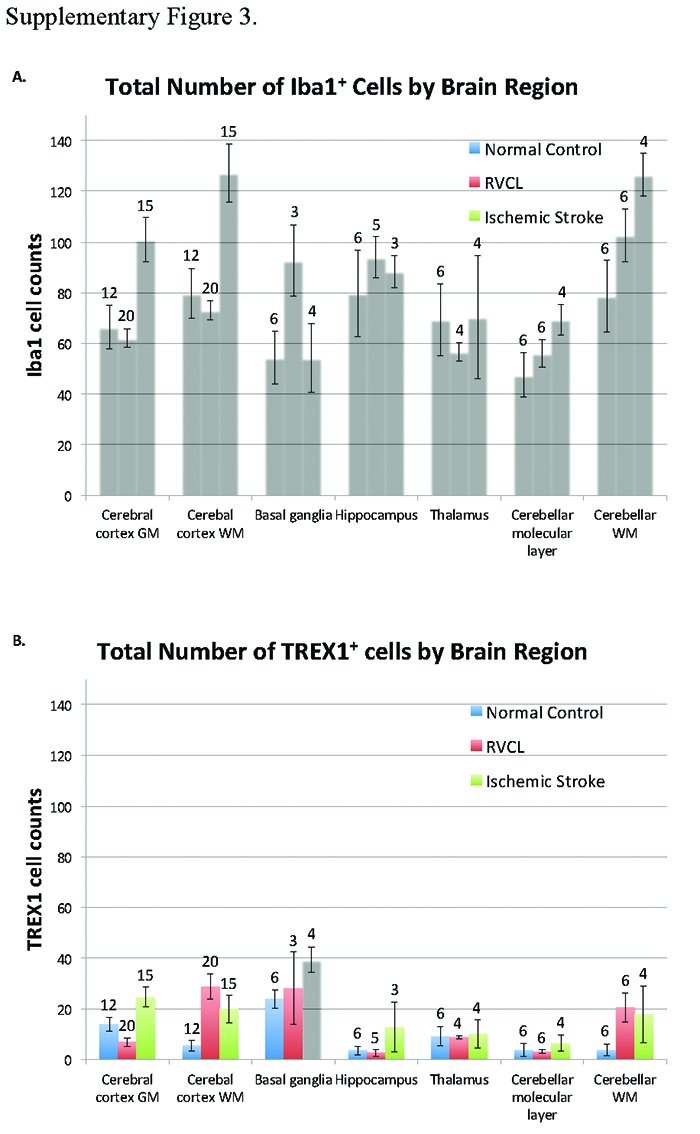

Supplement: Supplementary file 3 — Figure S3. Absolute cell counts for Iba1 and TREX1 by brain region in normal controls and cases of RVCL and ischemic stroke. The cell counts for Iba1 (A) and TREX1 (B) for specific areas of the brain in normal controls (blue bars), cases with RVCL (red bars) and cases with ischemic stroke (green bars). Sections quantified in RVCL and ischemic stroke were taken from undamaged tissue. The number of tissue sections included for each observation is noted above each column. White matter (WM); Gray matter (GM). Data are presented as mean ± SEM. [file BPA-28-806-s003.tif]

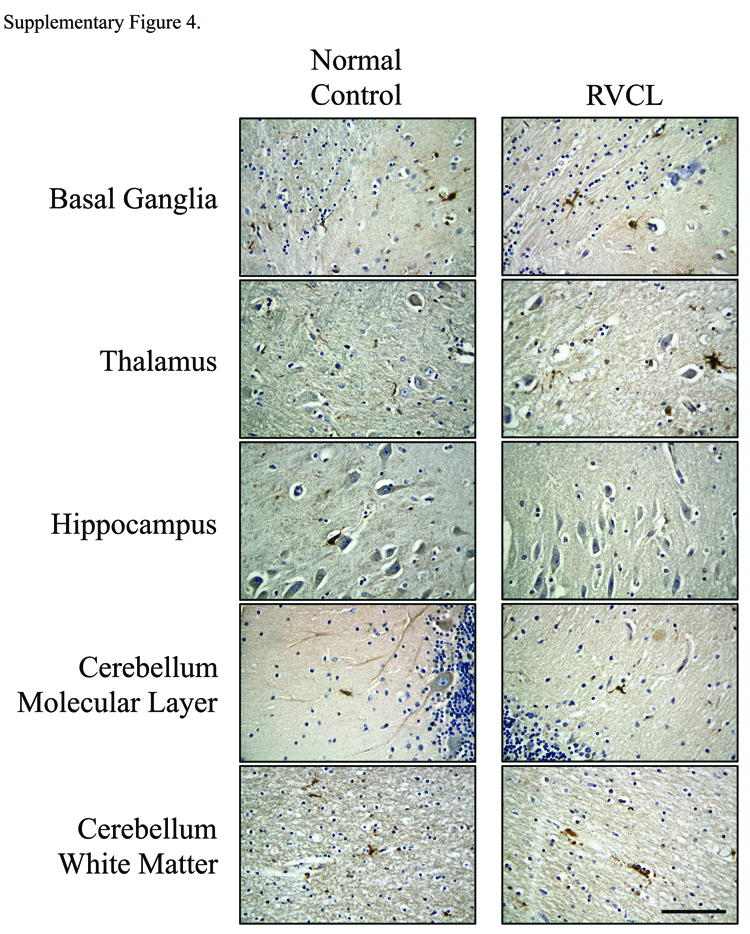

Supplement: Supplementary file 4 — Figure S4. Representative immunohistochemical staining for TREX1 (brown) in normal controls (left panel) and undamaged tissue in RVCL cases (right panel) in specified brain regions. Nuclei are counterstained with hematoxylin (blue). Scale bar represents 100 μm. [file BPA-28-806-s006.tiff]

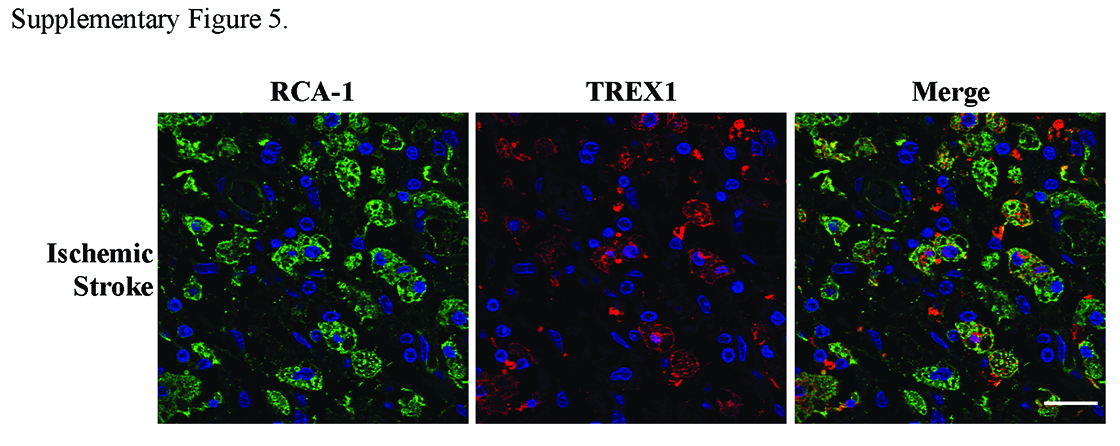

Supplement: Supplementary file 5 — Figure S5. TREX1 cells are ameboid microglia or infiltrating peripheral macrophages along the edges of ischemic lesions. Dual staining of formalin‐fixed, paraffin‐embedded human brain tissue from a case of ischemic stroke with RCA‐1 (left panel, green), a microglial/macrophage and endothelial cell marker, and anti‐TREX1 (red). Nuclei are counterstained with TO‐PRO‐3 (blue). Scale bar represents 28 μm. [file BPA-28-806-s002.tif]
