# Supplementary material for: TREX1 is expressed by microglia in normal human brain and increases in regions affected by ischemia
Source: Brain Pathol. 2018 Oct 10;28(6):806–21. doi: 10.1111/bpa.12626 (PMC6404532; doi:10.1111/bpa.12626)
Supplement: Supplementary file 6 — Table S1. Summary of the tissue sections analyzed. [file BPA-28-806-s001.docx]

Supplementary Table 1. Summary of the tissue sections analyzed.

|  |  | Frontal | Occipital | Basal Ganglia | Hippocampus | Thalamus | Cerebellum | Other |
| --- | --- | --- | --- | --- | --- | --- | --- | --- |
| Normal controls | 1 | 1 | 1 | 1 | 1 | 1 | 1 | 0 |
|  | 2 | 1 | 1 | 1 | 1 | 1 | 1 | 0 |
|  | 3 | 1 | 1 | 1 | 1 | 1 | 1 | 0 |
|  | 4 | 1 | 1 | 1 | 1 | 1 | 1 | 0 |
|  | 5 | 1 | 1 | 1 | 1 | 1 | 1 | 0 |
|  | 6 | 1 | 1 | 1 | 1 | 1 | 1 | 0 |
| RVCL | 1 | 2 | 2 | 1 | 1 | 1 | 1 | 2 |
|  | 2 | 2 | 0 | 0 | 0 | 0 | 0 | 2 |
|  | 3 | 2 | 1 | 1 | 1 | 1 | 1 | 0 |
|  | 4 | 1 | 2 | 1 | 1 | 1 | 1 | 7 |
|  | 5 | 2 | 2 | 1 | 2 | 2 | 3 | 5 |
| Ischemic stroke | 1 | 3 | 1 | 1 | 1 | 1 | 1 | 0 |
|  | 2 | 1 | 2 | 1 | 1 | 1 | 2 | 7 |
|  | 3 | 3 | 1 | 1 | 1 | 1 | 1 | 3 |
|  | 4 | 1 | 3 | 2 | 1 | 1 | 1 | 0 |
